# PhytoCluster: a generative deep learning model for clustering plant single-cell RNA-seq data

**DOI:** 10.1007/s42994-025-00196-6

**Published:** 2025-02-20

**Authors:** Hao Wang, Xiangzheng Fu, Lijia Liu, Yi Wang, Jingpeng Hong, Bintao Pan, Yaning Cao, Yanqing Chen, Yongsheng Cao, Xiaoding Ma, Wei Fang, Shen Yan

**Affiliations:** 1 State Key Laboratory of Crop Gene Resources and Breeding, Institute of Crop Science, Chinese Academy of Agricultural Sciences, Beijing, 100081 China; 2 School of Chinese Medicine, Hong Kong Baptist University, Hong Kong SAR, 999077 China; 3 College of Information and Management Science, Henan Agricultural University, Zhengzhou, 450002 China; 4 Washington University in St. Louis, St. Louis, 63130 USA

**Keywords:** scRNA-seq, Deep learning, Cellular heterogeneity, Latent features, Clustering

## Abstract

Single-cell RNA sequencing (scRNA-seq) technology enables a deep understanding of cellular differentiation during plant development and reveals heterogeneity among the cells of a given tissue. However, the computational characterization of such cellular heterogeneity is complicated by the high dimensionality, sparsity, and biological noise inherent to the raw data. Here, we introduce PhytoCluster, an unsupervised deep learning algorithm, to cluster scRNA-seq data by extracting latent features. We benchmarked PhytoCluster against four simulated datasets and five real scRNA-seq datasets with varying protocols and data quality levels. A comprehensive evaluation indicated that PhytoCluster outperforms other methods in clustering accuracy, noise removal, and signal retention. Additionally, we evaluated the performance of the latent features extracted by PhytoCluster across four machine learning models. The computational results highlight the ability of PhytoCluster to extract meaningful information from plant scRNA-seq data, with machine learning models achieving accuracy comparable to that of raw features. We believe that PhytoCluster will be a valuable tool for disentangling complex cellular heterogeneity based on scRNA-seq data.

**Supplementary Information:**

The online version contains supplementary material available at 10.1007/s42994-025-00196-6.

## Introduction

Single-cell RNA-seq (scRNA-seq) has quickly become widely adopted in plants and medical research as an ultra-high-resolution and ultra-high-throughput transcriptome profiling technology (Wang et al. 2023; Yu et al. 2024; Zheng et al. 2023). For plant biology, scRNA-seq has become instrumental for studying complex cellular heterogeneity, cell type–specific gene expression, and tissue development, enhancing an understanding of plant growth, stress responses, and cell differentiation (Cervantes-Perez et al. 2023; Deng et al. 2024; Jean-Baptiste et al. 2019; Joung et al. 2024). By capturing gene expression patterns, at the single-cell level, scRNA-seq provides unique opportunities to uncover previously uncharacterized cell types and regulatory mechanisms critical for plant development and adaptation.

The efficacy of the methods used to cluster scRNA-seq data strongly influences the accuracy and quality of results from subsequent downstream analyses, including cell type identification, gene expression analysis, and trajectory inference. However, clustering analysis of plant scRNA-seq data faces numerous challenges. Unlike animal cells, plant cells have rigid cell walls that need to be enzymatically removed to release cells amenable for scRNA-seq, which may introduce technical bias and can potentially alters cellular states (Ryu et al. 2019). Furthermore, substantial batch effects are often observed between replicates, due to variations among independent samples and experimental conditions (Haghverdi et al. 2018). Furthermore the large number of genes analyzed by scRNA-seq leads to datasets with dimensions equal to the number of expressed genes. This high dimensionality often causes the distances between data points (cells) to become similar, a phenomenon known as the “curse of dimensionality” (Boileau et al. 2020; Carangelo et al. 2022; Qiu et al. 2024). Therefore, precise clustering of scRNA-seq data is crucial for accurate interpretation and extraction of meaningful information.

Many clustering algorithms are generic, such as principal component analysis (PCA), which projects data into a lower-dimensional space; likewise, *k*-means clustering assigns samples to the closest centroid. These methods often fail to capture the nonlinear and complex structures of high-dimensional scRNA-seq data (Kiselev et al. 2019). More advanced methods, such as Scanpy (Wolf et al. 2018) and Seurat (Satija et al. 2015), have become popular for such large datasets, but they are limited by the suboptimal performance of the Louvain algorithm on smaller datasets (Ronhovde and Nussinov 2010). Deep learning methods offer a promising alternative by extracting nonlinear cellular features and improving clustering efficiency (Chen et al. 2020; Tian et al. 2021). For instance, the scVI model, based on hierarchical bayesian deep learning, demonstrated superior noise tolerance and clustering accuracy (Lopez et al. 2018). TripletCell is a powerful and robust tool for annotating cell types, although its accuracy in predicting subpopulations of plant cells requires improvements (Liu et al. 2023). Additionally, most of these tools were developed primarily for studying mammalian cells and might not have fully captured the characteristics of plant scRNA-seq data. Advancing the development of tools tailored to plant data would enable more precise analysis, allowing for deeper exploration of cellular differentiation.

To address these issues, we developed PhytoCluster, a generative deep neural network–based method. PhytoCluster integrates two model components: a variational autoencoder (VAE) framework and a Gaussian mixture model (GMM). PhytoCluster is an unsupervised deep learning method that requires no labeled data for training. We compared PhytoCluster to advanced methods used for scRNA-seq analysis, including PCA, scVI, Scanpy, and Seurat. PhytoCluster achieved better clustering performance than these other models in different scenarios, using simulations and real datasets. We validated the ability of PhytoCluster to denoise scRNA-seq data using an autoencoder that extracts latent features to guide the clustering step. By training machine learning models on latent features, we show that PhytoCluster effectively extracted valuable information from scRNA-seq data, improving the accuracy and effectiveness of clustering. Overall, PhytoCluster should serve as a powerful tool for improving our understanding of plant cell types and their characteristics within complex biological systems.

## Results

### Overview of PhytoCluster

**Fig. 1:**
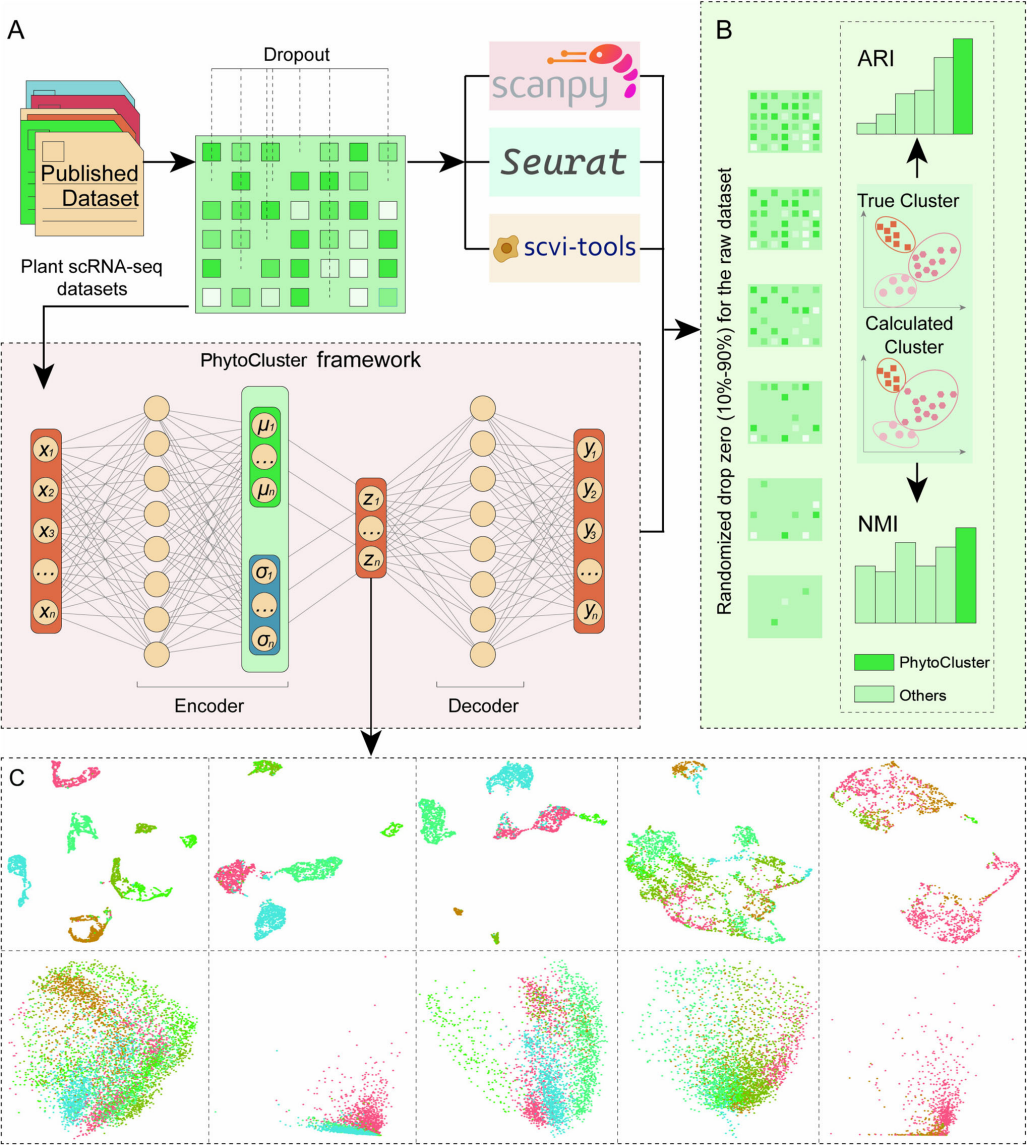
Overview of the PhytoCluster framework. **A** The PhytoCluster model receives gene expression vectors and compresses the raw expression data in an encoder to infer the latent features that help retain the signal after data compression. The decoder then reconstructs the original gene expression matrix of each cell based on these latent features. **B** The robustness of PhytoCluster and other methods to data corruption was tested by randomly dropping values in the raw dataset to zero. **C** *t*-SNE plots after processing of single-cell datasets by different methods

An overview of PhytoCluster is shown in Fig. 1. As the input for PhytoCluster, the obtained scRNA-seq data are first preprocessed, including the removal of low-abundance genes and cells, as well as data normalization. PhytoCluster is an unsupervised deep learning model that combines encoder and decoder structures to extract biologically meaningful latent features from scRNA-seq data, offering a holistic view of cellular variability. These latent features are low-dimensional representations of the transformed data, capturing its essential features and underlying patterns. Serving as compact, noise-reduced representations, they enhance clustering accuracy and facilitate downstream analysis. PhytoCluster initializes the model with parameters obtained from an autoencoder, iteratively optimizes the clustering objective function, and reconstructs the input data from the latent features using its decoder. By employing metrics like Normalized Mutual Information (NMI) and Adjusted Rand Index (ARI) values and leveraging *t*-distributed stochastic neighbor embedding (*t*-SNE) visualizations, we comprehensively validated PhytoCluster against existing approaches across simulated and real datasets, demonstrating its superior capability in clustering scRNA-seq data, as explained below.

### Benchmarking PhytoCluster in simulation datasets

**Fig. 2:**
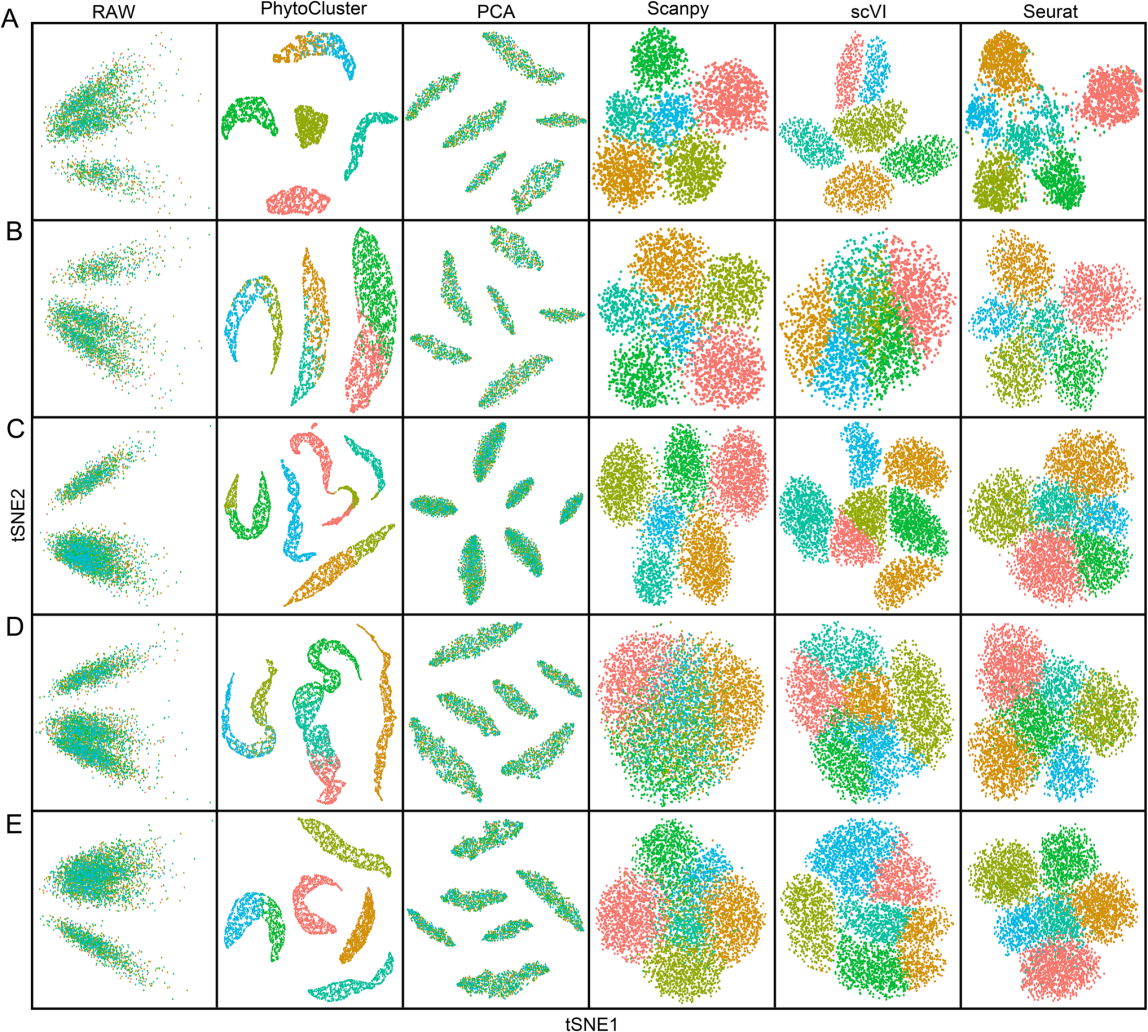
Feature embedding and clustering on simulated datasets. **A**–**E** *t*-distributed stochastic neighbor embedding (*t*-SNE) plots of the raw data and the features extracted by PhytoCluster, principal component analysis (PCA), Scanpy, scVI, and Seurat in the simulated datasets. To ensure consistency, PhytoCluster, PCA, Scanpy, scVI, and Seurat were used to drop the data to only 10 dimensions before applying *t*-SNE, whereas the raw data were directly processed by *t*-SNE without dimensionality reduction

Clustering of scRNA-seq data is a critical task that influences the discrimination of cell populations and subpopulations. PhytoCluster addresses this stage of the analysis by leveraging latent features that encapsulate essential biological information while minimizing noise and redundancy. To evaluate the clustering performance of PhytoCluster, we assessed its ability to recover expression values from simulated scRNA-seq data under various noise distributions, namely the gamma, gaussian, and negative binomial distributions. Notably, there were substantial differences between plant and animal scRNA-seq data (Fig. S1). For example, compared to mouse datasets, rice scRNA-seq data exhibit lower sparsity, posing challenges such as redundant information and noise interference. To address these differences, we simulated datasets based on the distribution characteristics of plant scRNA-seq data (Table S1). We consistently observed superior performance for PhytoCluster as compared to other clustering methods like PCA, Scanpy, scVI, and Seurat across all noise types (Fig. 2). By using latent features, PhytoCluster demonstrated robust clustering performance across all simulation datasets, highlighting its adaptability to diverse data distributions.

### Benchmarking PhytoCluster in real datasets

**Fig. 3:**
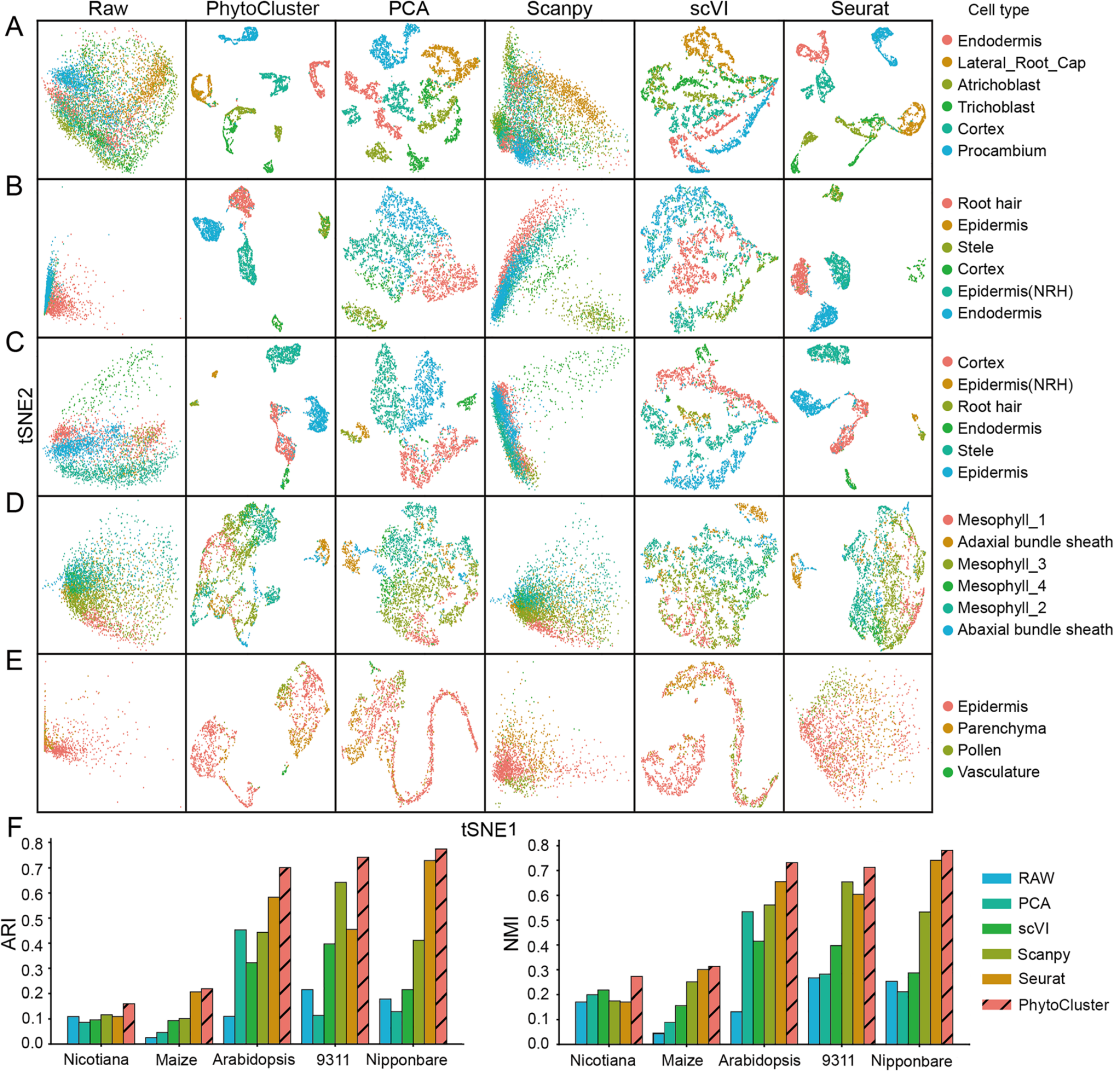
Feature embedding and clustering on real datasets. **A**–**E** *t*-SNE plots of the raw data and the features extracted from PhytoCluster, PCA, Scanpy, scVI, and Seurat for the Arabidopsis dataset (**A**), the rice *indica* (93–11) dataset (**B**), the rice *japonica* (Nipponbare) dataset (**C**), the maize lead dataset (**D**), and the *Nicotiana attenuata* corolla dataset (**E**). **F** Adjusted Rand Index (ARI) and Normalized Mutual Information (NMI) values for each of the real datasets following clustering with PhytoCluster and the other methods

We next evaluated the performance of PhytoCluster on several real datasets. To this end, we applied PhytoCluster to five datasets obtained from various of plant species through multiple sequencing platforms. Summaries of these real scRNA-seq datasets are presented in Table S2–6. Initially, we trained PhytoCluster using an Arabidopsis (*Arabidopsis thaliana*) dataset and conducted a benchmarking analysis. The dataset consisted of scRNA-seq profiles from 6000 Arabidopsis root cells (GSE152766), produced on the 10X Genomics platform, corresponding to six cell types (Shahan et al. 2022). PhytoCluster used the latent features of this dataset to effectively capture key information, resulting in clear separation of cell types (Fig. 3A and F). Compared to other methods, PhytoCluster achieved the highest scores in terms of NMI and ARI values (NMI = 0.732; ARI = 0.701), outperforming the second-best ranked method, Seurat (NMI = 0.655; ARI = 0.583), by 7.7% and 11.8%, respectively (Table S7).

PhytoCluster also exhibited competitive performance with ARI values reaching 0.742, 0.775, 0.160, and 0.219 on the other plant scRNA-seq datasets derived from *indica* rice roots (93–11) (Liu et al. 2021), *japonica* rice roots (Nipponbare)(Liu et al. 2021), *Nicotiana attenuata* corolla (Kang et al. 2022), and maize (*Zea mays*) leaves (Bezrutczyk et al. 2021), respectively. PhytoCluster continued to demonstrate an advantage as assessed by the NMI metric. Specifically, PhytoCluster outperformed the runner-up model by 5.9%, 4.0%, 5.5%, and 1.2%, for each of these datasets (Fig. 3B–F and Table S7). However, it should be noted that when the number of cells is severely limited, clustering performance may be poor. For instance, for maize leaves, the ARI and NMI scores were only 21.9% and 31.3% (Fig. 3D). Similarly, in the *Nicotiana attenuata* corolla dataset, the parenchyma, pollen, and vasculature cell subpopulations contained fewer cells than the epidermis cells (Fig. 3E). These imbalances explain the relatively poor performance of PhytoCluster on the *N. attenuata* and maize datasets. In summary, PhytoCluster, leveraging its latent feature extraction capability, effectively organizes disordered data in the original space, clearly separates distinct cell subpopulations, and accurately clusters similar cells, even in challenging real datasets.

### Evaluating the robustness of PhytoCluster on highly corrupted data

We evaluated the robustness of PhytoCluster to data sparsity by randomly setting scRNA-seq values in the raw dataset to zero. Specifically, we randomly masked the number of genes detected in each cell with varying dropout rates (10–90%) and assessed the clustering accuracy of PhytoCluster and other tools using ARI and NMI values. To optimize model performance, we selected the Arabidopsis, *indica* (93–11), and *japonica* (Nipponbare) datasets, all of which have balanced cell numbers. By extracting latent features, PhytoCluster preserved the biological integrity of the data and maintained high clustering performance even under severe corruption.

**Fig. 4:**
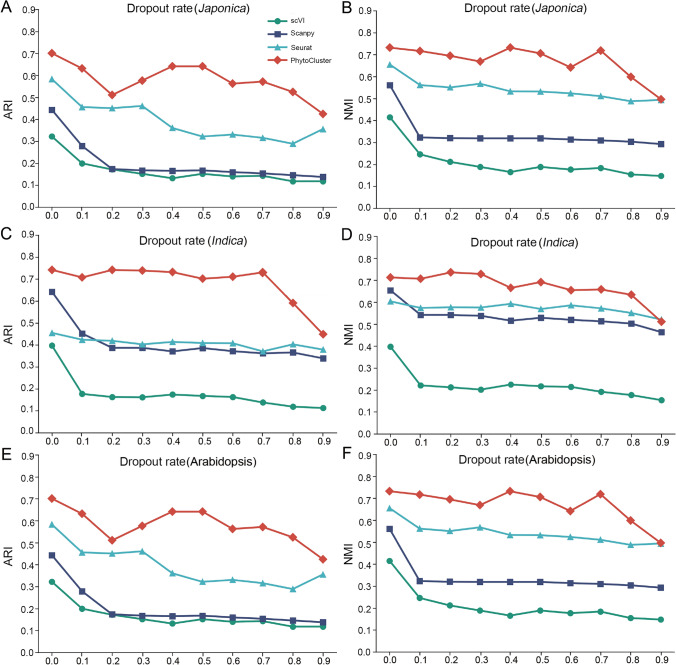
Consequences of data corruption on preserving the original data structure. **A**, **B** ARI values (**A**) and NMI values (**B**) for the four methods at different corruption levels (0.1–0.9) using the rice *japonica* (Nipponbare) dataset. **C**, **D** ARI values (**C**) and NMI values (**D**) for the four methods at different corruption levels (0.1–0.9) using the rice *indica* (93–11) dataset. **E**, **F** ARI values (**E**) and NMI values (**F**) for the four methods at different corruption levels (0.1–0.9) on the Arabidopsis dataset

For example, on the *japonica* (Nipponbare) dataset, even with a 90% dropout rate, PhytoCluster maintained high clustering performance, achieving an NMI value of 0.633 and a ARI value of 0.590 (Fig. 4A and B). Both Scanpy and Seurat also exhibited robustness under similar conditions, although their performance declined more than that of PhytoCluster (Fig. 4A and B). On the *indica* (93–11) dataset, PhytoCluster yielded an ARI value of 0.731 at a 70% dropout rate, representing a modest decrease from 0.742 with the raw data (Fig. 4C and D), while Seurat and Scanpy experienced substantial drops in ARI values, from 0.455 to 0.371 and from 0.642 to 0.362, respectively. Similarly, on the Arabidopsis dataset, PhytoCluster outperformed other methods across all corruption levels (Fig. 4E and F). These results demonstrate that the latent features extracted by PhytoCluster effectively preserve the essential biological patterns in scRNA-seq data, making it highly robust to data corruption.

### Latent features preserve the true structure of the real dataset

We extended our study beyond evaluating the ability of PhytoCluster to extract latent features, remove noise, and accurately represent the distribution of scRNA-seq data in real datasets, by further examining the performance of these latent features in machine learning tasks. Specifically, PhytoCluster was applied to the Arabidopsis, *indica* (93–11), and *japonica* (Nipponbare) datasets, extracting 10 latent features from the input data. These features provided a noise-reduced, compact representation of gene expression profiles from individual cells while preserving true biological variation. We then tested these latent features by training four machine learning models (Support Vector Machines [SVM], Random Forest Classifier [RFC], Extreme Gradient Boosting [XGBoost], and Light Gradient Boosting Machine [LightGBM]) using five-fold cross-validation (Table S8). We evaluated the models with four metrics: accuracy, precision, recall, and F1 measure. To ensure a fair comparison, the dataset was split into training (80%) and testing (20%) subsets, and all models were executed on the same test set.

**Fig. 5:**
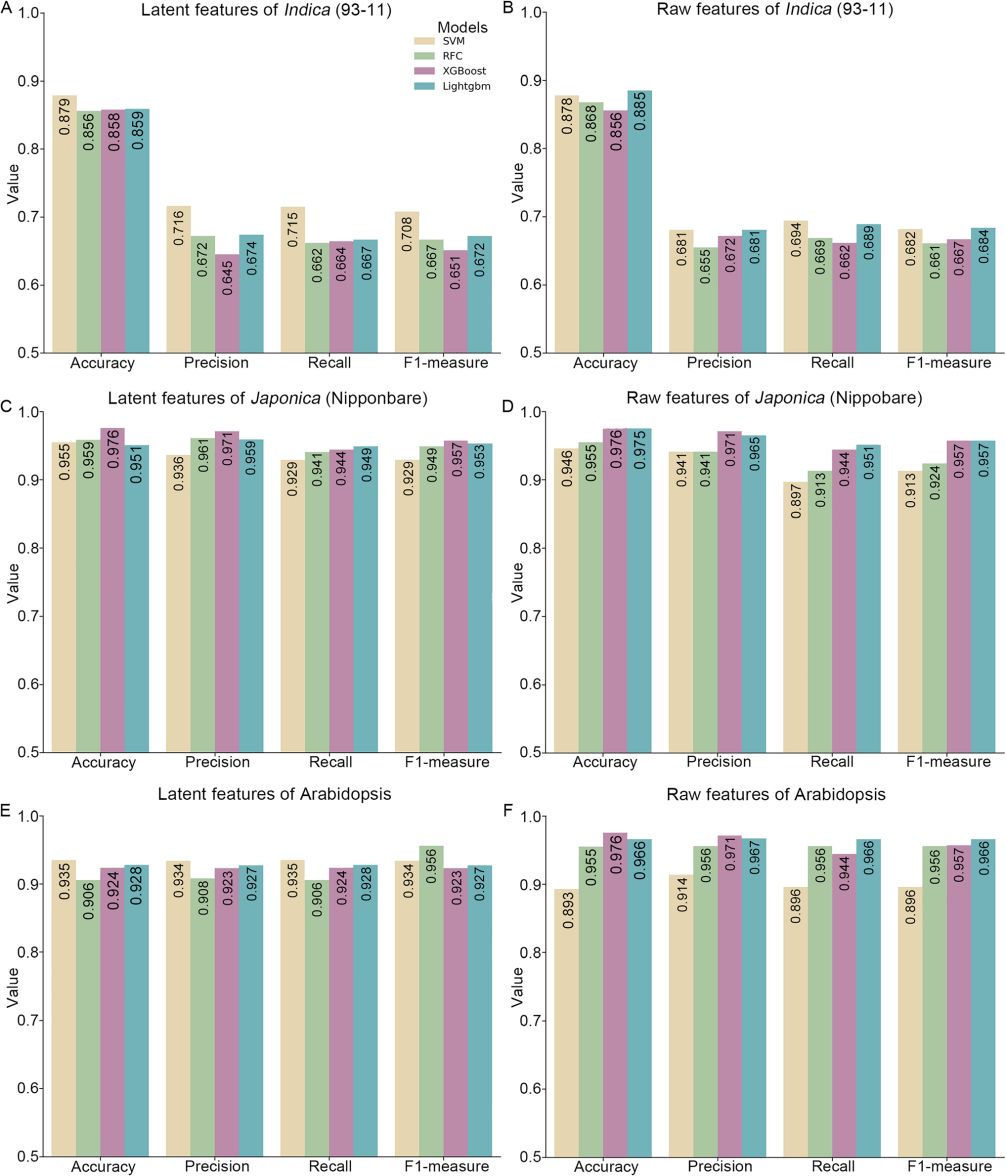
Evaluation of the performance of latent and raw features by machine learning algorithms. **A**, **B** Predictive performance of latent features (**A**) and raw features (**B**) extracted by PhytoCluster, using one of four machine learning methods on the rice *indica* (93–11) dataset. **C**, **D** Predictive performance of latent features (**C**) and raw features (**D**) using one of four machine learning methods, based on the rice *japonica* (Nipponbare) dataset. **E**, **F** Predictive performance of latent features (**E**) and raw features (**F**) using one of the four machine learning methods on the Arabidopsis dataset

We clearly noticed the advantages of latent features in this analysis. For the *indica* (93–11) dataset, models trained on latent features achieved high prediction accuracies: 0.879 for SVM, 0.856 for RFC, 0.858 for XGBoost, and 0.859 for LightGBM, comparable to their performance on raw features (Fig. 5A, B and Table S9). However, latent features provided improved performance in key metrics for the *japonica* (Nipponbare) dataset, where machine learning models showed better accuracy, precision, and recall compared to the use of raw features (Fig. 5C, D and Table S10). Similarly, in the Arabidopsis dataset, latent features yielded results that were comparable or superior to raw features (Fig. 5E, F and Table S11). These results highlight the critical role of latent features in enhancing the overall performance of PhytoCluster. By reducing data complexity while preserving key biological information, PhytoCluster emerges as a powerful and efficient tool for scRNA-seq data analysis.

## Discussion

Studies at the single-cell level have gradually become more common in plant science and are already generating large amounts of data. In this work, we developed the PhytoCluster framework to cluster scRNA-seq data by extracting latent features. We extensively tested PhytoCluster using simulated and real datasets from various plants (Arabidopsis, rice, *N. attenuata*, and maize) generated with different scRNA-seq protocols. Comparison to other advanced analytical methods demonstrated that PhytoCluster achieved high clustering accuracy across all datasets. The framework is based on the PyTorch platform and leverages GPU-accelerated computation.

Notably, PhytoCluster demonstrated a performance advantage in preserving the original structure of the data. By randomly dropping data at varying ratios, PhytoCluster performed best compared to other methods, retaining the original structure more effectively when data corruption levels were below approximately 60%. Additionally, machine learning models trained on the latent features achieved performance comparable to those trained on raw features. These results indicate that PhytoCluster can effectively simulate and remove various types of noise and artifacts in scRNA-seq data, including dropout, uniform random noise, Gaussian noise, gamma noise, and negative binomial noise.

However, this study is not without limitations. While PhytoCluster performed well across many scRNA-seq datasets, challenges remain in scenarios where cell numbers of different cell subpopulations are highly imbalanced. Furthermore, in our analysis of real data, the training time of the PhytoCluster model showed no advantage over other methods, leading to higher computational costs. In the future, we aim to enhance data collection and optimize the model structure to improve training efficiency. Overall, we demonstrate that PhytoCluster provides robust clustering capabilities for scRNA-seq data and effectively facilitates downstream analysis. It empowers researchers to explore specific gene functions and understand the influence of external factors on plant growth and development at the single-cell level. Additionally, PhytoCluster should help breeders in identifying candidate genes to improve crop traits and designing more effective breeding strategies.

## Materials and methods

### Datasets and preprocessing

The Arabidopsis dataset was derived from root tip cells of the Arabidopsis (*Arabidopsis thaliana*) wild-type accession Columbia (Col-0) and of two mutant lines (*short root-2* and *scarecrow-4*), collected from the primary root tips of 5–7-day-old independently grown seedlings (Shahan et al. 2022) (Table S2). The rice dataset was derived from protoplasts isolated from the tips of crown roots (5 mm in length) from 3-day-old seedlings of two major rice cultivars—Nipponbare (Nip, *japonica* group) and 93–11 (*indica* group)—which are key parental lines for breeding in Asia (Liu et al. 2021) (Tables S3 and S4). The coyote tobacco (*Nicotiana attenuata*) dataset was obtained from protoplasts isolated from the corolla limbs and throat cups of *N. attenuata* flowers at three time points: Zeitgeber time 8 (ZT8, 8 h after light onset), ZT12, and ZT1622, grown under 16-h light/8-h dark conditions at 26 °C with a ± 2 °C variation (Table S5). The maize dataset was derived from protoplasts isolated from the distal portion of fully differentiated leaves (6-cm segment from leaf 2) of V2 stage maize seedlings (Bezrutczyk et al. 2021) (Table S6). The scRNA-seq dataset for mouse spinal cord injury was obtained from the study by Li et al. (2022a). Following preprocessing by the same methods as Li et al. (2022a) a mouse dataset was generated comprising expression data from 6000 single cells, including endothelial cells, astrocytes, microglia, neurons, oligodendrocytes, and pericyte cells (Table S12).

For the Arabidopsis dataset, genes with no counts in any cell were removed, and cells were filtered iteratively to exclude low-quality cells based on mitochondrial gene expression enrichment (> 5% of total UMI counts). For the rice dataset, cells with fewer than 200 detected genes and genes detected in fewer than 20 cells were excluded. Similarly, for the *Nicotiana attenuata* dataset, cells with fewer than 200 expressed genes were discarded. For the maize dataset, the filtering criteria were set as follows: percent.pt < 4 andpercent.mt < 0.75 andnFeature_RNA > 1800, and nFeature_RNA < 7000.

### PhytoCluster encoder module

The encoder in the Variational Autoencoder (VAE) was designed to map the high-dimensional input data X into a lower-dimensional latent space. This procedure involves a neural network that generates two vectors as output: the mean μ and the logarithm of the variance $\text{log}{\sigma }^{2}$, which parameterize the approximate posterior distribution $q(z|x)$. Specifically, given an input $x$, the encoder network computes:

1 $$\mu = f_{\mu } \left( x \right)$$

2 $$\log \sigma^{2} = f_{\sigma } \left( x \right)$$

where ${f}_{\mu }$ and ${f}_{\sigma }$ are neural networks. These outputs are used to sample the latent variable *z* through the reparameterization trick, ensuring that the gradient can propagate through the sampling procedure. The reparameterization trick was defined as:

3 $$z=\mu +\sigma \odot\upepsilon$$

where $\epsilon \sim \mathcal{N}(0,I)$ is sampled from a standard normal distribution and $\sigma =\text{exp}\left(\frac{\text{log}{\sigma }^{2}}{2}\right)$.

### PhytoCluster decoder module

The decoder in the VAE aims to reconstruct the input data X from the latent variables *z*. It is a neural network that maps *z* back to the data space, producing a distribution over the possible reconstructions $\widehat{x}$. Formally, the decoder network ${g}_{\theta }$ parameterizes the likelihood $p(x|z)$. Given a latent variable *z*, the decoder computes:

4 $$\widehat{x}={g}_{\theta }\left(z\right)$$

where ${g}_{\theta }$ is a neural network. The objective of the decoder is to maximize the likelihood of the observed data under the generated distribution, ensuring that the reconstructed data $\widehat{x}$ are as close as possible to the original input $x$.

### PhytoCluster Gaussian mixture model (GMM) module

GMM has been widely used for unsupervised model-based clustering of complex data. In this study, we considered the latent matrix $\text{H}={\left[{\text{h}}_{\text{i}\text{d}}\right]}_{\text{n}\times {\text{p}}_{\text{e}}}$ where $i=\text{1,2},\dots ,n$ and $d= 1,2,\dots , {p}_{\text{e}}$. Each row ${\text{h}}_{\text{i}}$ represents one cell and ${p}_{\text{e}}$ is the latent embedding size. The goal of using a GMM is to cluster ${\text{h}}_{\text{i}}$ into *K* components or clusters. The probability density function of *K*-component GMM is given by

5 $${f}_{\text{gmm}}\left(\mathbf{h};\theta \right)=\sum_{k=1}^{K} {\pi }_{k}\phi \left(\mathbf{h};{\mu }_{k},{\Sigma }_{k}\right) $$

where ${\mu }_{k}$ and ${\Sigma }_{k}$ are mean vectors and covariance matrices of mixture components, respectively. For $k=\text{1,2},\dots ,K$, ${\pi }_{k}>0$ is the weight of the *k*^th^ mixture component restricted to $\sum_{k=1}^{K} {\pi }_{k}=1$. The GMM can be parameterized by $\theta =({\pi }_{1},\dots ,{\pi }_{k},{{\varvec{\mu}}}_{1},\dots ,{{\varvec{\mu}}}_{\mathbf{k}},{\Sigma }_{1},\dots ,{\Sigma }_{k})$. Here, it can be estimated using the well-known statistical algorithm EM (El Assaad et al. 2016; Garriga et al. 2016; Uykan 2021; Tsumoto et al. 2022), with *K*-means (Yu et al. 2018; Malik and Tuckfield 2019; Sinaga and Yang 2020) as the initialization clustering method. In the proposed method, we considered $\sum_{\mathbf{k}}$ as a diagonal covariance.

### Latent feature extraction

The latent feature extraction in PhytoCluster was implemented through a VAE framework, which compresses high-dimensional scRNA-seq data into a biologically meaningful latent space. The encoder in PhytoCluster consists of a two-layer neural network with Rectified Linear Unit (ReLU) activation functions designed to progressively decrease the dimensionality of the input data. The dimension-reduced data are then mapped to a latent layer with 10 neurons, representing the dimensionality of the latent feature space. To prevent overfitting, dropout regularization was applied, enabling the encoder to generalize to unseen data and avoid reliance on specific input patterns. The VAE framework generates two key parameters, the mean $(\mu )$ and log variance $(\text{log}{\sigma }^{2})$, which define the approximate posterior distribution of the latent variables $z$. The latent variables are sampled using the reparameterization trick, as shown in Eq. 3.

### PhytoCluster clustering optimization

Hui et al. (2020) proposed GMM-VGAE as an attributed graph clustering model. In this algorithm, the number of cell clusters *k* will be either pre-determined based on the prior information (e.g. the number of labels in the labeled data) or estimated based on the unlabeled data. Given a feature matrix, an initial clustering assignment is conducted using a GMM to obtain the estimates of cluster parameters ${\pi }_{k},{ \mu }_{k}$, ${\sigma }_{k}$ that are the probability, mean, and standard deviation of cluster *k*, respectively, such that $\pi \in {\mathbb{R}}_{+}^{K}\text{ and}\sum_{k=1}^{K} {\pi }_{k}=1$. These parameters are later merged to obtain the combined designed loss function for the framework. The model is then trained and optimized by maximizing the modified loss function, which can be defined as

6 $${\mathcal{L}}_{\text{CL}}={E}_{q(h,k|x,\alpha )}\left[\text{log}\frac{p\left(\alpha ,h,k\right)}{q\left(h,k|x,\alpha \right)}\right]$$

where $p\left(\alpha ,h,k\right)=p\left(\alpha |h\right)p\left(h|k\right)p\left(k\right)\text{ and }q\left(h,k|x,\alpha \right)=q\left(h|x,\alpha \right)q\left(k|x,\alpha \right)$ is the variational posterior. To optimize the clustering (CL) ${\mathcal{L}}_{\text{CL}}$ using standard stochastic gradient methods, a stochastic gradient variational Bayes estimator and the reparameterization trick were used, as proposed for ELBO loss in VAE (Kingma and Welling 2013). The ${\mathcal{L}}_{\text{CL}}$ can also be divided into reconstruction loss ${\mathcal{L}}_{\text{REC}}$ and Kullback–Leibler (KL) divergence loss $q\left(h|x,\alpha \right)=\mathcal{N}\left(h;\widetilde{\mu },{\widetilde{\sigma }}^{2}I\right)$. Once the training is completed by maximizing Eq. (1), the latent representation *h* can be obtained. The clustering assignments can be obtained by

7 $$q\left( {k{|}x,a} \right) = p\left( {k{|}h} \right) = \frac{{p\left( k \right)p\left( {h{|}k} \right)}}{{\mathop \sum \nolimits_{{k^{\prime} = 1}}^{k} p\left( {k{^{\prime}}} \right)p\left( {h{|}k^{\prime}} \right)}}{ }$$

### Simulated scRNA-seq datasets

Four groups of scRNA-seq datasets were simulated using Splatter (Zappia et al. 2017) (Table S1). Building on this, different distributions of noise were added to generate an additional four datasets, with noise following a random uniform, gaussian, gamma, or negative binomial distribution. The implementation involved using np.random to generate noise from different distributions, which was then added to the scRNA-seq dataset. All values were rounded to the nearest integer, and any negative values were set to zero.

### Machine learning

PhytoCluster-extracted latent and raw features from scRNA-seq were used to train the XGBoost (Chen and Guestrin 2016), SVM (Chang and Lin 2011), Lightgbm (Yan et al. 2021), and RFC (Scornet 2016) base models and compare their predictions. The machine learning model was implemented using the Python package “scikit-learn”. To optimize the performance of the four machine learning models, the grid search function was used to adjust the hyperparameters to optimal values.

### Clustering

The *K*-means clustering method from the Python scikit-learn package was used to cluster the input scRNA-seq data based on the extracted features.

### Visualization

The *t*-SNE method from the Python scikit-learn package was used to project the raw data or latent features into two dimensions.

### Evaluation metrics

To benchmark the various methods on different datasets, the Adjusted Rand Index (ARI) (Li et al. 2022b; Qian et al. 2024) and Normalized Mutual Information (NMI) (Qiu et al. 2024; Yu et al. 2023; Tian et al. 2021) were used to quantify the accuracy of each clustering result. The four classic metrics were used to quantify the performance of model predictions were accuracy, recall, precision, and F1 measure (Ma et al. 2024; Wang et al. 2021, 2024; Lin et al. 2024; Yan and Wang 2022).

8 $$ARI= \frac{ {\sum }_{\mathcalligra{i}\mathcalligra{j}}\left(\begin{array}{c}{n}_{\mathcalligra{i}\mathcalligra{j}}\\ 2\end{array}\right)-\left[ {\sum }_{\mathcalligra{i}}\left(\begin{array}{c}{a}_{\mathcalligra{i}}\\ 2\end{array}\right){\sum }_{\mathcalligra{j}}\left(\begin{array}{c}{b}_{\mathcalligra{j}}\\ 2\end{array}\right)\right]/\left(\begin{array}{c}n\\ 2\end{array}\right)}{\frac{1}{2}\left[{\sum }_{\mathcalligra{j}}\left(\begin{array}{c}{a}_{\mathcalligra{i}}\\ 2\end{array}\right)+{\sum }_{\mathcalligra{j}}\left(\begin{array}{c}{b}_{\mathcalligra{j}}\\ 2\end{array}\right)\right]-\left[{\sum }_{\mathcalligra{j}}\left(\begin{array}{c}{a}_{\mathcalligra{i}}\\ 2\end{array}\right)+{\sum }_{\mathcalligra{j}}\left(\begin{array}{c}{b}_{\mathcalligra{j}}\\ 2\end{array}\right)\right]/\left(\begin{array}{c}n\\ 2\end{array}\right) }$$

where ${n}_{\mathcalligra{i}\mathcalligra{j}}$ represents the number of cells shared between cluster $i$ in the clustering result and cell type $j$ from the true labels; ${a}_{\mathcalligra{i}}$ denotes the number of cells in cluster $i$; ${b}_{\mathcalligra{j}}$ is the number of cells in cell type $j$, and $n$ refers to the total cell count. The ARI value was used to compare the clustering results of the integrated data with the predefined cell types. ARI values range from 0 to 1, where higher values indicate a greater similarity between the clustering results and the true cell type labels.

9 $$\text{NMI}=2\times \frac{{\sum }_{\mathcalligra{i}\mathcalligra{j}}\frac{{n}_{\mathcalligra{i}\mathcalligra{j}}}{n}\text{log}\left(\frac{n\times {n}_{\mathcalligra{i}\mathcalligra{j}}}{{a}_{\mathcalligra{i}}\times {b}_{\mathcalligra{j}}}\right)}{ {\sum }_{\mathcalligra{i}}\frac{{a}_{\mathcalligra{i}}}{n}\text{log}\left(\frac{n}{{a}_{\mathcalligra{i}}}\right)+{\sum }_{\mathcalligra{j}}\frac{{b}_{\mathcalligra{j}}}{n}\text{log}\left(\frac{n}{{b}_{\mathcalligra{j}}}\right) }$$

where the notations are the same as for ARI. NMI values range from 0 to 1 and higher values also indicate higher similarities between the clustering results and true cell types.

10 $$\text{Accuracy}=\frac{TP+TN}{TP+TN+FP+FN}$$

11 $$\text{Recall}=\frac{TP}{TP+FN}$$

12 $$\text{Precision}=\frac{TP}{TP+FP}$$

13 $$F1\text{ measure}=\frac{2*\left(\text{precision}*\text{recall}\right)}{\text{precision}+\text{recall}}$$

where $TP, TN, FP,\text{ and }FN$ represent the numbers of true positives, true negatives, false positives and false negatives, respectively.

## Supplementary Information

Below is the link to the electronic supplementary material.

Supplementary file1 (DOCX 172 KB)

## Data Availability

Several published scRNA-seq datasets and five simulated datasets were used in this study, all accessible through the accession numbers provided in the original publications and below. Simulated datasets are available from https://gitee.com/lihaicheng7003/cluster.git. The Arabidopsis dataset (GSE152766) was downloaded from https://www.ncbi.nlm.nih.gov/geo/query/acc.cgi?acc=GSE152766. The rice *indica* (93–11) and *japonica* (Nipponbare) datasets (GSE146035) are available at https://www.ncbi.nlm.nih.gov/geo/query/acc.cgi?acc=GSE146035. The *Nicotiana attenuata* corolla dataset (GSE193464) can be obtained from https://www.ncbi.nlm.nih.gov/geo/query/acc.cgi?acc=GSE193464. The maize leaf dataset (GSE157759) is available from https://www.ncbi.nlm.nih.gov/geo/query/acc.cgi?acc=GSE157759. The scRNA-seq dataset of crush-injured adult mouse spinal cord that support the findings of this study are available in figshare under the link https://doi.org/10.6084/m9.figshare.17702045. The code used in this study is also available at GitHub (https://github.com/Llana-168/PhytoCluster).
