# Supplementary material for: PhytoCluster: a generative deep learning model for clustering plant single-cell RNA-seq data
Source: aBIOTECH. 2025 Feb 20;6(2):189–201. doi: 10.1007/s42994-025-00196-6 (PMC12237842; doi:10.1007/s42994-025-00196-6)
Supplement: Supplementary file 1 — Supplementary file1 (DOCX 172 KB) [file 42994_2025_196_MOESM1_ESM.docx]

**PhytoCluster: a generative deep learning model for clustering plant single-cell RNA-seq data**

Hao Wang^1,†^, Xiangzheng Fu^2,†^, Lijia Liu^1,†^, Yi Wang^1,4^, Jingpeng Hong^1,3^, Bintao Pan^1^, Yaning Cao^1^, Yanqing Chen^1^, Yongsheng Cao^1^, Xiaoding Ma^1,*^, Wei Fang^1,*^, Shen Yan^1,*^

^1^ State Key Laboratory of Crop Gene Resources and Breeding, Institute of Crop Sciences, Chinese Academy of Agricultural Sciences, Beijing 100081, China

^2^ School of Chinese Medicine, Hong Kong Baptist University, Hong Kong, SAR 999077, China

^3^ College of Information and Management Science, Henan Agricultural University, Zhengzhou 450002, China

^4^ Washington University in St. Louis, St. Louis, 63130, The United States

* Corresponding authors: Shen Yan, Email: yanshen@caas.cn; Wei Fang, Email: fangwei@caas.cn; Xiaoding Ma, Email: maxiaoding@caas.cn.

†These authors contributed equally to this work.

**Supplementary Notes**

**Supplementary Table 1.** Summary of simulated scRNA-seq datasets with non-dropout noise used in this study.

**Supplementary Table 2.** Composition of cell subpopulations of the scRNA-seq dataset for Arabidopsis.

**Supplementary Table 3.** Composition of cell subpopulations of the scRNA-seq dataset for the rice *indica* cultivar 93-11.

**Supplementary Table 4.** Composition of cell subpopulations of the scRNA-seq dataset for the rice *japonica* cultivar Nipponbare.

**Supplementary Table 5.** Composition of cell subpopulations of the scRNA-seq dataset of *Nicotiana attenuata* corolla.

**Supplementary Table 6.** Composition of cell subpopulations of the scRNA-seq dataset for maize leaves.

**Supplementary Table 7.** Differences in clustering accuracy between PhytoCluster and runner-up models for various datasets.

**Supplementary Table 8.** Best hyperparameter values of the four models selected by grid search.

**Supplementary Table 9.** Performance of latent and raw features of the rice *indica* cultivar 93-11 dataset on different machine learning models. Testing dataset.

**Supplementary Table 10.** Performance of latent and raw features of the rice *japonica* (Nipponbare) dataset with different machine learning models. Testing dataset.

**Supplementary Table 11.** Performance of latent and raw features of the Arabidopsis dataset with different machine learning models. Testing dataset.

**Supplementary Table 12.** Composition of cell subpopulations of the scRNA-seq dataset for mouse spinal cord.

**Supplementary Fig. 1** Differences between the animal (mouse) and plant (rice cultivar Nipponbare) scRNA-seq data.

**Supplementary Table 1.** Summary of simulated scRNA-seq datasets with non-dropout noise used in this study.

| Dataset | Number of genes | Number of cells | Groups | Noise type | Parameter |
| --- | --- | --- | --- | --- | --- |
| Dataset 1 | 10,000 | 3,000 | 6 | None | None |
| Dataset 2 | 10,000 | 3,000 | 6 | Uniform | Low = –5, high = 15 |
| Dataset 3 | 10,000 | 5,000 | 6 | Gamma | shape = 2, scale = 0.5 |
| Dataset 4 | 10,000 | 5,000 | 6 | Gaussian | loc=0, scale=6 |
| Dataset 5 | 10,000 | 5,000 | 6 | Negative binomial | nb_mean = −2, nb_dispersion = 0.5 |

**Supplementary Table 2.** Composition of cell subpopulations of the scRNA-seq dataset for Arabidopsis.

| Cell type | Number of cells |
| --- | --- |
| Endodermis | 1,000 |
| Lateral root cap | 1,000 |
| Atrichoblast | 1,000 |
| Trichoblast | 1,000 |
| Cortex | 1,000 |
| Procambium | 1,000 |

**Supplementary Table 3.** Composition of cell subpopulations of the scRNA-seq dataset for the rice *indica* cultivar 93-11.

| Cell type | Number of cells |
| --- | --- |
| Cortex | 1,000 |
| Root hair | 337 |
| Epidermis (non-root hair [NRH]) | 200 |
| Endodermis | 200 |
| Stele | 1,000 |
| Epidermis | 1,000 |

**Supplementary Table 4.** Composition of cell subpopulations of the scRNA-seq dataset for the rice *japonica* cultivar Nipponbare.

| Cell type | Number of cells |
| --- | --- |
| Cortex | 1,000 |
| Root hair | 131 |
| Epidermis (non-root hair [NRH]) | 121 |
| Endodermis | 211 |
| Stele | 1,000 |
| Epidermis | 1,000 |

**Supplementary Table 5.** Composition of cell subpopulations of the scRNA-seq dataset of *Nicotiana attenuata* corolla.

| Cell type | Number of cells |
| --- | --- |
| Epidermis | 1,127 |
| Parenchyma | 388 |
| Pollen | 168 |
| Vasculature | 35 |

**Supplementary Table 6.** Composition of cell subpopulations of the scRNA-seq dataset for maize leaves.

| Cell type | Number of cells |
| --- | --- |
| Mesophyll (1) | 327 |
| Mesophyll (2) | 1,024 |
| Mesophyll (3) | 1,201 |
| Mesophyll (4) | 292 |
| Abaxial bundle sheath | 225 |
| Adaxial bundle sheath | 365 |

**Supplementary Table 7.** Differences in clustering accuracy between PhytoCluster and runner-up models for various datasets.

| Datasets | Metrics | PhytoCluster accuracy | Runner-up model | Improvement |
| --- | --- | --- | --- | --- |
| Arabidopsis | ARI | 70.1% | 58.3% | 11.8% |
| *indica* rice (93-11) |  | 74.2% | 64.2% | 10.0% |
| *japonica* rice (Nipponbare) |  | 77.5% | 72.9% | 4.6% |
| *Nicotiana attenuata* corolla |  | 16.0% | 11.6% | 4.4% |
| Maize leaves |  | 21.9% | 20.6% | 1.3% |
| Arabidopsis | NMI | 73.2% | 65.5% | 7.7% |
| *indica* rice (93-11) |  | 71.3% | 65.4% | 5.9% |
| *japonica* rice (Nipponbare) |  | 78.1% | 74.1% | 4.0% |
| *Nicotiana attenuata* corolla |  | 27.3% | 21.8% | 5.5% |
| Maize leaves |  | 31.3% | 30.1% | 1.2% |

**Supplementary Table 8** The optimal hyperparameter values of the four models selected by grid search.

| **Model** | **Hyperparameter** | **Values tested by grid search** |
| --- | --- | --- |
| SVM | kernel | 'rbf', 'sigmoid' |
|  | gamma | 'scale', 'auto' |
|  | C | 0.01, 0.1, 1, 5, 10 |
| RFC | max_depth | 3, 5, 7, 10 |
|  | min_samples_split | 2, 5, 10 |
|  | n_estimators | 10, 50, 100, 200 |
| XGBoost | max_depth | 3, 5, 7, 10 |
|  | min_child_weight | 1, 3, 5 |
|  | n_estimators | 10, 50, 100, 200 |
|  | learning_rate | 0.1, 0.01, 0.001 |
| Lightgbm | learning_rate | 0.1, 0.01, 0.001 |
|  | n_estimators | 10, 50, 100, 200 |
|  | num_leaves | 2, 5, 20, 30, 50, 60 |
|  | feature_fraction | 0.6, 0.7, 0.8 |

**Supplementary Table 9.** Performance of latent and raw features of the *indica* rice (93-11) dataset using different machine learning models. Testing dataset.

| Model | Features | Accuracy | Precision | Recall | F1-measure |
| --- | --- | --- | --- | --- | --- |
| SVM | Latent features | 87.9% | 71.6% | 71.5% | 70.8% |
| RFC |  | 85.6% | 67.2% | 66.2% | 66.7% |
| XGBoost |  | 85.8% | 64.5% | 66.4% | 65.1% |
| Lightgbm |  | 85.9% | 67.4% | 66.7% | 67.0% |
| SVM | Raw features | 87.8% | 68.1% | 69.4% | 68.2% |
| RFC |  | 86.8% | 65.5% | 66.9% | 66.1% |
| XGBoost |  | 85.6% | 67.2% | 66.2% | 66.7% |
| Lightgbm |  | 88.5% | 68.1% | 68.9% | 68.4% |

**Supplementary Table 10.** Performance of latent and raw features for the *japonica* rice (Nipponbare) dataset using different machine learning models. Testing dataset.

| Model | Features | Accuracy | Precision | Recall | F1-measure |
| --- | --- | --- | --- | --- | --- |
| SVM | Latent features | 95.5% | 93.6% | 92.9% | 92.9% |
| RFC |  | 95.9% | 96.1% | 94.1% | 94.9% |
| XGBoost |  | 97.6% | 97.1% | 94.4% | 95.7% |
| Lightgbm |  | 95.1% | 95.9% | 94.9% | 95.3% |
| SVM | Raw features | 94.6% | 94.1% | 89.7% | 91.3% |
| RFC |  | 95.5% | 94.1% | 91.3% | 92.4% |
| XGBoost |  | 97.6% | 97.1% | 94.4% | 95.7% |
| Lightgbm |  | 97.5% | 96.5% | 95.1% | 95.7% |

**Supplementary Table 11.** Performance of latent and raw features for the Arabidopsis dataset using different machine learning models. Testing dataset.

| Model | Features | Accuracy | Precision | Recall | F1-measure |
| --- | --- | --- | --- | --- | --- |
| SVM | Latent features | 93.5% | 0.93.4% | 0.93.5% | 0.93.4% |
| RFC |  | 90.6% | 0.90.8% | 0.90.6% | 0.95.6% |
| XGBoost |  | 92.4% | 0.92.3% | 0.92.4% | 0.92.3% |
| Lightgbm |  | 92.8% | 0.92.7% | 0.92.8% | 0.92.7% |
| SVM | Raw features | 89.3% | 0.91.4% | 0.89.6% | 0.89.6% |
| RFC |  | 95.5% | 0.95.6% | 0.95.6% | 0.95.6% |
| XGBoost |  | 97.6% | 0.97.1% | 0.94.4% | 0.95.7% |
| Lightgbm |  | 96.6% | 0.96.7% | 0.96.6% | 0.96.6% |

**Supplementary Table 12.** Composition of cell subpopulations of the scRNA-seq dataset for mouse spinal cord.

| Cell type | Number of cells |
| --- | --- |
| Endothelial | 1,000 |
| Astrocyte | 1,000 |
| Microglia | 1,000 |
| Neuron | 1,000 |
| Oligodendrocyte | 1,000 |
| Pericyte | 1,000 |

**
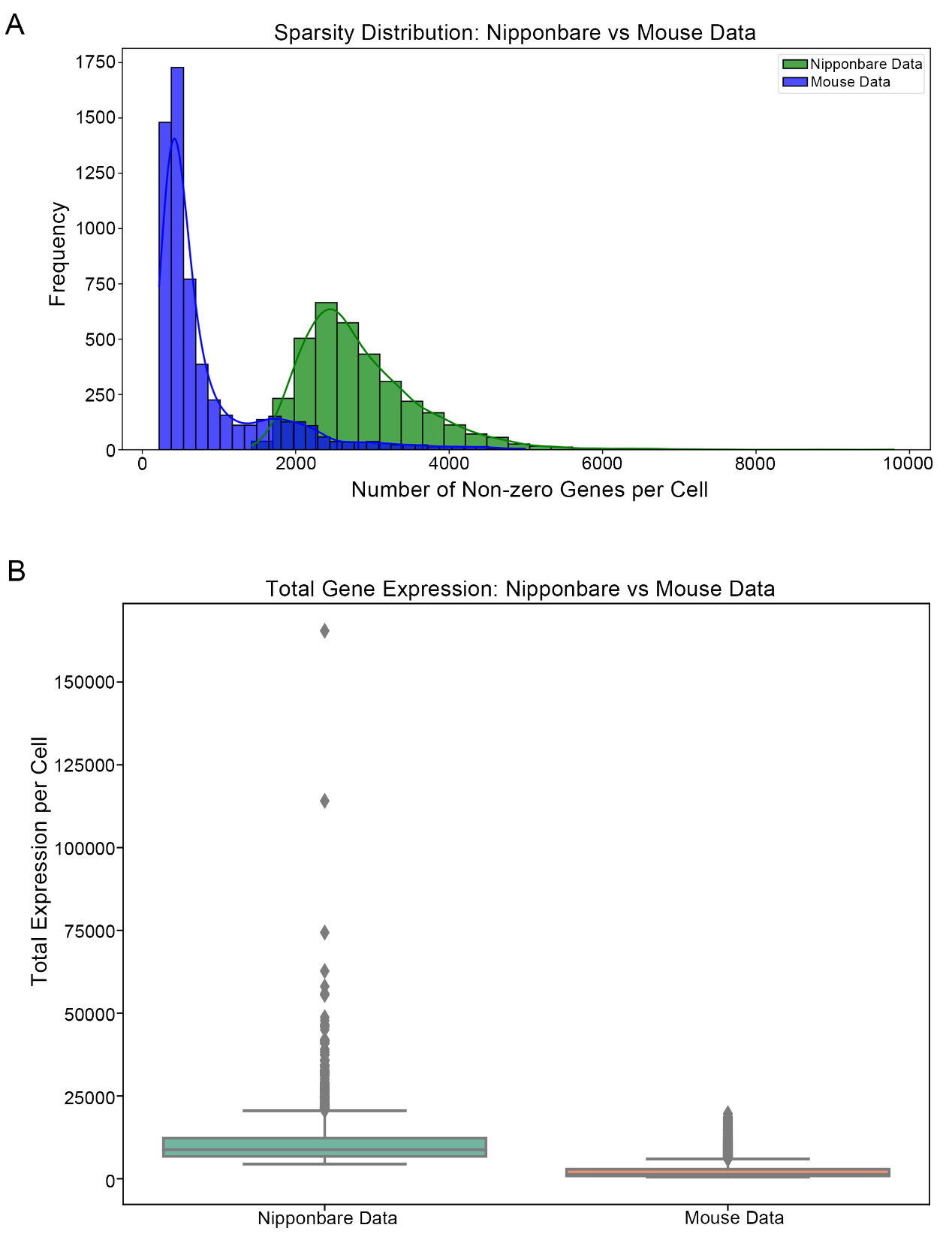
**

**Supplementary Fig. 1** Differences between the animal (mouse) and plant (Nipponbare) scRNA-seq data. (A) Histogram of the number of non-zero genes per cell for the Nipponbare (green) and mouse (blue) datasets. (B) Boxplot of total gene expression per cell in the Nipponbare (left) and mouse (right) datasets.
